# Genetic Association Analysis for Relative Growths of Body Compositions and Metabolic Traits to Body Weights in Broilers

**DOI:** 10.3390/ani11020469

**Published:** 2021-02-10

**Authors:** Ying Zhang, Hengyu Zhang, Yunfeng Zhao, Xiaojing Zhou, Jie Du, Runqing Yang

**Affiliations:** 1 College of Animal Science and Veterinary Medicine, Heilongjiang Bayi Agricultural University, Daqing 163319, China; yingzhang@byau.edu.cn; 2 Heilongjiang Provincial Key Laboratory of Efficient Utilization of Feed Resources and Nutrition Manipulation in Cold Region, Daqing 163319, China; 3 Department of Information and Computing Science, Heilongjiang Bayi Agricultural University, Daqing 163319, China; zhanghengyu555@sohu.com (H.Z.); zhouxiaojing7924@126.com (X.Z.); 4 Research Centre for Aquatic Biotechnology, Chinese Academy of Fishery Sciences, Beijing 100141, China; zhaoyf@cafs.ac.cn (Y.Z.); dujie_cafs@163.com (J.D.)

**Keywords:** allometry, QTL, random regression model, body composition, metabolic trait

## Abstract

**Simple Summary:**

The relative growth of body components and metabolic traits relative to body weights are phenotypically characterized using joint allometric scaling models, and random regression models (RRMs) are constructed to map quantitative trait loci (QTLs) for allometries of body compositions and metabolic traits in broilers. Prior to statistically inferring the QTLs for the allometric scalings, the QTL candidates in RRMs are obtained by rapidly shrinking most of marker genetic effects to zero with the LASSO technique. Referred to as real joint allometric scaling models, statistical utility of the so-called LASSO-RRM mapping method is demonstrated by computer simulation analysis. Using the F2 population by crossing broiler × Fayoumi, we formulate optimal joint allometric scaling models of fat, shank weight (shank-w) and liver as well as thyroxine (T4) and glucose (GLC) to body weights. For body compositions, a total of 9 QTLs, including 4 additive and 5 dominant, were detected to control the allometric scalings of fat, shank-w and liver to body weights; while for metabolic traits, total 10 QTLs, were mapped to govern the allometries of T4 and GLC to body weights, among which 6 QTLs were of dominant genetic effect. The detected QTLs or highly linked markers can be used to regulate relative growths for meat quality traits to body weight in marker-assisted breeding of broilers.

**Abstract:**

In animal breeding, body components and metabolic traits always fall behind body weights in genetic improvement, which leads to the decline in standards and qualities of animal products. Phenotypically, the relative growth of multiple body components and metabolic traits relative to body weights are characterized by using joint allometric scaling models, and then random regression models (RRMs) are constructed to map quantitative trait loci (QTLs) for relative grwoth allometries of body compositions and metabolic traits in chicken. Referred to as real joint allometric scaling models, statistical utility of the so-called LASSO-RRM mapping method is given a demonstration by computer simulation analysis. Using the F2 population by crossing broiler × Fayoumi, we formulated optimal joint allometric scaling models of fat, shank weight (shank-w) and liver as well as thyroxine (T4) and glucose (GLC) to body weights. For body compositions, a total of 9 QTLs, including 4 additive and 5 dominant QTLs, were detected to control the allometric scalings of fat, shank-w, and liver to body weights; while a total of 10 QTLs of which 6 were dominant, were mapped to govern the allometries of T4 and GLC to body weights. We characterized relative growths of body compositions and metabolic traits to body weights in broilers with joint allometric scaling models and detected QTLs for the allometry scalings of the relative growths by using RRMs. The identified QTLs, including their highly linked genetic markers, could be used to order relative growths of the body components or metabolic traits to body weights in marker-assisted breeding programs for improving the standard and quality of broiler meat products.

## 1. Introduction

In animal linkage analysis, the resource populations, although genetically designed, did not satisfy strict back cross (BC) and F2 structures as used in plant breeding because of the difficulty to produce complete homozygous parents. Previously, with pseudo-BC or F2 population of multiple small families, the association of genetic markers with target traits has been statistically inferred by regressing phenotypic value variances to identity by descent at markers between pairwise siblings [1]. However, the regression method is inappropriate for multiple large families and populations with complex pedigree because of too many pairwise relatives. As an alternative, the random models [2], where genetic effects of markers were regarded as random, were proposed to map QTLs. It has been demonstrated that the statistical power of maximum likelihood method is higher than that of the regression method in QTL mapping [3]. Linear mixed models (LMMs) have been used to map QTLs in structured populations with multiple families which take QTL (genetic marker) effects as fixed and consider random confounding effects caused by complex pedigree [4]. However, genome-wide mixed model association study undertakes heavy computational burden. Therefore, some new algorithms [5,6,7,8,9] have been subsequently proposed to fit LMMs more simply. Instead of one test at a time, multi-marker mixed models have been jointly analyzed with stepwise regression analysis [10] and a LMM-LASSO [11].

Compared with absolute growth, relative growth refers to the growth or development of a certain biological trait, relative to changes in another trait of interest, like whole body size or body weight. Different tissues have different growth rates and allometric scaling is used to describe the relative growth rate of partial body size to the whole-body [12,13]. Allometric growth has been comprehensively studied in animal and plant science [14]. The Allometric scaling model was firstly proposed by Huxley (1932) and was used to describe the allometric relationships of relative growth of certain biological trait to whole body size [15]. Considering the internal genetic correlations among multiple partial body compositions, the joint static allometry scaling model [16] was developed to simultaneously assess the allometric scalings of different biological traits to the whole-body size. In terms of allometric growth genetics, analyses of gene mapping of allometric scalings among multiple phenotypic traits have been implemented by embedding simple allometric functions into additive genetic effects of LMM [17,18,19,20,21], and into genotypic effect items of genetic model to detect QTLs [22,23,24]. Nevertheless, these mapping methods are all developed based on a single QTL model, which has lower statistical power in mapping QTLs when allometric scalings are genetically regulated by multiple significant QTLs [20].

Here we firstly characterized the allometric growths of multiple body components and metabolic traits relative to body weight by using a joint allometric scaling model, then established an random regression model (RRM) [25] to fit the genetic effects of microsatellite markers and minor polygenes derived from the pedigree on the studied allometric traits. Before statistical inference of mapping the QTLs of the allometric traits with the RRM, we obtained a relatively small number of non-zero effect QTL candidates with LASSO technique [26] that efficiently shrank most genetic effects of minor markers to zero in the over-saturated models of marker effects. Hence the method we used for QTL mapping is named “LASSO-RRM”. This mapping method showed its efficiency when applied to detect the QTLs for the allometries of body compositions and metabolic traits in Fayoumi chicken. Meanwhile, simulation studies were performed to demonstrate the statistical utility of our proposed LASSO-RRM method.

## 2. Materials and Methods

### 2.1. Population and Phenotypes

An F2 resource population was constructed by crossing sire lines from a broiler breeder with dam lines from genetically distinct and highly inbred Fayoumi chicken [27]. 325 chickens from 20 families, five to 25 individuals per family, were used for mapping experiment. The total number of genotyped microsatellite markers was 190, covering 3835-cM-long genome regions including 19 autosomes, the Z chromosome and two additional linkage groups [28]. Whole body weight (BW) and 8 metabolic traits involving glucose (GLC), insulin (INS), INS:glucagon (IGR), insulin-like growth factor I (IGFI), insulin-like growth factor II (IGFII), lactate (LCT), thyroxine (T4) and triiodothyronine (T3) concentrations in plasma and also 9 body composition measures, including abdominal fat weight (Fat), breast muscle weight (BMW), drumstick weight (Drumstick), heart weight (Heart), liver weight (Liver), shank weight (Shank-w), spleen weight (Spleen), Drumstick length (Drumstick-L) and shank length (Shank-L) were measured at the age of 8 weeks [29,30].

### 2.2. Joint Allometric Scaling Model

Joint static allometric scaling model [31] of these body compositions or metabolic traits $x_{i}\;(i=1,\;2,\;\cdots ,\;m)$ to body weights *y* is defined as:

(1) $$y=\beta_{0}{x_{1}}^{\beta_{1}}{x_{2}}^{\beta_{2}}\cdots {x_{m}}^{\beta_{m}}$$

where $\beta_{0}$ is an intercept, $\beta_{j}(j=1,\cdots ,m)$ is partial scaling exponent of the *j*-th body composition or metabolic trait to body weight.

For the convenience to estimate model parameters, model (1) is transformed into:

(2) $$\ln y=\ln\beta_{0}+\beta_{1}\ln x_{1}+\beta_{2}\ln x_{2}+\cdots +\beta_{m}\ln x_{m}$$

For the linear regression model (2), we established an optimal joint static allometric scaling model by stepwise regression analysis, filtering out significant partial allometric scalings (significant $\beta_{j}$) of body compositions or metabolic traits related to body weight with phenotypic records described in Section 2.1. In the optimized joint static allometric scaling model, if partial allometric scaling of body composition or metabolic trait is greater than 1, then the relative growth of this trait is lower than that of body weight, and vice versa. If the value of partial allometric scaling is equal to 1, it means that the growth rate of body composition or metabolic trait and body weight is equivalent.

### 2.3. LMM for Multiple QTLs

We detected QTLs for body weights in the same F_2_ population described in Section 2.1 with linkage analysis method for genetic markers. When the density of microsatellite markers is lower-moderate, a number of pseudo-markers will be evenly placed by 1 *cM* within the intervals of neighboring markers on the genetic linkage map. Besides additive and dominant genetic effects, polygenic genetic effects can also be estimated by the pedigree of multiple full-sib families. The Equation (3) is a classic animal model was used to map QTLs for body weights of broilers:

(3) $$y_{i}=\sum_{j=1}^{p}h_{ij}b_{j}+\sum_{j=1}^{q}(z_{ij}a_{j}+w_{ij}d_{j})+g_{i}+e_{i}$$

where *y*_i_ is body weight of the *i*-th broiler, *p* is the number of fixed effects for sex and hatch, $b_{j}$ is the *j*-th fixed effect of sex and hatch, *a_j_* and *d_j_* are the *j*-th additive and dominant genetic effects, respectively, of *q* genotyped markers and inserted pseudo-markers, *h_ij_*, *z_ij_* and *w_ij_* are the indicator variables of *b_j_*, *a_j_* and *d_j_*, *g_i_* is the polygene effect, and *e_i_* is the residual error of the *i*-th broiler. It is generally assumed that $g_{i}∼N(0,\;\sigma_{g}^{2})$ with $\sigma_{g}^{2}$ is polygenic variance, and $e_{i}∼N\left(0,\sigma_{e}^{2}\right)$ with $\sigma_{e}^{2}$ is residual variance.

### 2.4. RRM for Multiple QTLs

By nesting the optimal joint allometric scaling model (2) into the items of the fixed effects, marker genetic effects, and polygenic effects of model (3), we establish an RRM to simultaneously map the QTLs of the allometries of body compositions or metabolic traits relative to body weights:

(4) $$\ln y_{i}=\sum_{j=1}^{p}h_{ij}b_{j}^{T}x_{i}+\sum_{j=1}^{q}[z_{ij}a_{j}x_{i}+w_{ij}d_{j}x_{i}]+g_{i}x_{i}+e_{i}$$

where $x_{i}={\left[\begin{array}{cccc} 1 & \ln x_{1i} & \cdots & \ln x_{mi} \end{array}\right]}^{T}$.

Let $y=\left[\begin{array}{cccc} \ln y_{1} & \ln y_{2} & \cdots & \ln y_{n} \end{array}\right]$, $X=\left[\begin{array}{cccc} x_{1} & x_{2} & \cdots & x_{n} \end{array}\right]$, $h_{j}=\left[\begin{array}{cccc} h_{1j} & h_{2j} & \cdots & h_{nj} \end{array}\right]$, $z=\left[\begin{array}{cccc} z_{1} & z_{2} & \cdots & z_{n} \end{array}\right]$, $w=\left[\begin{array}{cccc} w_{1} & w_{2} & \cdots & w_{n} \end{array}\right]$, $G={\left[\begin{array}{cccc} g_{1} & g_{2} & \cdots & g_{n} \end{array}\right]}^{T}$ and $e={\left[\begin{array}{cccc} e_{1} & e_{2} & \cdots & e_{n} \end{array}\right]}^{T}$ for all *n* broilers in the population. In the notation of matrices, model (4) is rewritten as:

(5) $$y=\sum_{j=1}^{p}h_{j}b_{j}+\sum_{j=1}^{q}(za_{j}+wd_{j})+XG+e$$

The model satisfies:

(6) $$E(y|\theta )=\sum_{j=1}^{p}h_{j}b_{j}+\sum_{j=1}^{q}\left(za_{j}+wd_{j}\right)\;V(y)=A⊗P+I\sigma_{e}^{2}$$

where $\theta =\left[\begin{array}{cccc} \theta_{0} & \theta_{1} & \cdots & \theta_{m} \end{array}\right]$ with θ∈*b*, *a*, *d* or *g,* A is relationship matrix calculated with pedigree, P is genetic covariance matrix for multiple allometric scalings and I is identity matrix. The statistical analysis was conducted by using DMU software [32].

### 2.5. Statistical Inference of QTLs

Since the number of microsatellite markers is far greater than that of subjects in the population, the existing methods for solving LMM are not able to statistically infer multiple QTLs in the oversaturated RRM. Considering those sparse QTLs detected always with linkage analysis, we reduce the number of markers to a relatively small number with major effects by using LASSO technique implemented in R/glmnet [33]:

(7) $$a_{j}\;\mathrm{or}\;d_{j}=\arg\min\left\{\left[y-E(y|\theta )]{\left[y-E(y|\theta )\right]}^{T}+\lambda (|a_{j}|+|d_{j}|\right)\right\}$$

where λ is the tuning parameter.

As QTL candidates, the selected major effects are analyzed as the genetic effect item of marker in model (7). We estimate the variance components in the RRM with the QTL candidates by using the REML algorithm, and then statistically infer the QTLs in the following statistics:

(8) $$t_{kj}=\frac{\left|{\hat{a}}_{kj}\;or\;{\hat{d}}_{kj}\right|}{\sqrt{Var(a_{kj}\;or\;d_{kj})}}$$

where *k* = 1, 2, …, m and *j* = 1, 2, …, q.

Under a significance level of 5%, we use permutation test to determine the empirical critical value of declaring the significance of candidate QTL [34].

## 3. Results

### 3.1. Constructing Joint Allometric Scaling Models

By stepwise regression, we established the optimal joint static allometric scaling models for body compositions and metabolic traits to body weights, which are:

(9) $$\hat{y}=7.0518x_{1}^{0.1258}x_{2}^{0.5711}x_{3}^{0.1250}$$

for fat ($x_{1}$), shank-w ($x_{2}$) and liver ($x_{3}$), and

(10) $$\hat{y}=31.2988x_{1}^{0.1201}x_{2}^{-0.1116}$$

for T4 ($x_{1}$) and GLC ($x_{2}$), respectively. This indicated that the relative growth of fat, shank-w, liver, T4 and GLC were significantly associated with body weight among the phenotypic traits measured. Taking model (11) and (12) as sub-models of RRMs for multiple QTLs, we used the LASSO-RRM to map QTLs for allometric scalings (relative growth) of the three body compositions and the two metabolic traits to body weights as below.

### 3.2. Mapping QTLs for the Allometric Scalings of Body Compositions

Table 1 summarizes the QTLs of the allometric scalings of the three body compositions to body weights. Two additive and three dominant QTLs were detected for fat, of which the QTL on chromosome 27 fell on the microsatellite marker. For Shank-w, total three QTLs, including one additive and two dominant, were mapped on chromosome 6, 8 and 11. Only one dominant QTL for liver was found between markers LEI265 and LEI65 on chromosome 3. All QTLs had a positive impact on the allometries, no matter what body compositions analyzed or in what genetic modes.

### 3.3. Mapping QTLs for the Allometric Scalings of Metabolic Traits

Table 2 shows QTL mapping results for the allometric scalings of metabolic traits (T4 and GLC) with linkage analysis. Five QTLs were detected for each analyzed trait, two additive and three dominant. One negative additive QTL for T4 fell on the marker ADL144 on chromosome 4. The two additive QTLs had opposite genetic effects, while all dominant QTLs performed positive genetic effects on T4. Both additive QTLs positively influenced and all dominant QTLs negatively on GLC. One additive and two dominant QTLs were detected on chromosome 1 for GLC.

### 3.4. Simulation Analysis

We designed an F2 resource population of small families of different sizes with a function from the R/qtl package [35]. Three chromosomes, each 100 cM long, were simulated. 11, 21 and 101 codominant markers were distributed on every chromosome with marker intervals of 10, 5 and 1 *cM* on average, respectively. Based on linkage analysis for the allometries of body compositions to body weights, 8 allometric QTLs for the fat, liver and shank-w were put on the simulated chromosomes, whose positions and additive effects were given in Table 3. The logarithm of body weights was generated by the regression effects $\left[\begin{array}{c} b_{1}\\ b_{2} \end{array}\right]=\left[\begin{array}{ccc} 3.2123 & -3.1803\times 10^{-3} & -3.9772\times 10^{-5}\\ 3.3830 & -5.415\times 10^{-2} & -1.7252\times 10^{-2} \end{array}\right]$ for sex, the genetic covariance matrix $P=\left[\begin{array}{ccc} 9.4268 & 0.6888 & -3.0253\\ 0.6888 & 1.8840 & -0.7581\\ -3.0253 & -0.758 & 1.1309 \end{array}\right]\times 10^{-3}$ and residual variance $\sigma_{e}^{2}$ = 2.7 × 10^−2^. A marker was identified as the QTL if its test statistic was the maximum among its 20 closest neighbors and exceeded the threshold value. 200 repeated simulations were conducted to evaluate estimated parameters and statistical power to map QTLs. Under a significant level of 5%, the statistical power was defined as the percentage of the number of those simulations in which a significant QTL was detected.

Statistical power to detect QTLs and parameter estimates (standard deviations) are shown in Table 4 for the simulated data. For the same marker density, simulation results show the following general properties: (1) QTLs of large effects are easier to detect than those of small effects, that is, the statistical power to detect QTLs increases with the increase of QTL effect. (2) The statistical power and parameter estimation vary with the analyzed traits. (3) There is no obvious difference in statistical properties between pleiotropic and single-effect QTLs for the same traits. As the marker density increases, the statistical power and parameter estimation significantly improve. The maximum increment in statistical power is 54% in the simulations, which occurs at the second simulated QTL of the heart. In summary, it is necessary to extend the linkage analysis to genome-wide association analysis, to improve the mapping of QTLs for relative growths of partial body compositions or metabolic traits to whole body weights in animals.

## 4. Discussion

Allometric growth of animals has long been of interest to animal agricultural communities. Generally, ontogenetic, static and evolutionary allometries are the three types of allometries, of which the first two have been the subject of extensive recent research. An ontogenetic allometry describes the relative size of traits throughout the growth of an individual. A static allometry describes the relative size of traits, among individuals at the same developmental stage, within populations [36,37,38]. Obviously, the allometric phenotypes in our research belong to static allometry. The partial scaling exponent $\beta_{j}\;(j=1,\cdots ,m)$ in the static allometry model quantifies the different growth rates of related traits [16]. From our fitting results of the allometric model (11) and (12), the growth rates of body compositions and metabolic traits are much lower than whole body weight, which supports that with the development of animal breeding, the growth rate of body weight continues to improve in terms of genetic value, while genetic improvement of meat quality traits, as represented by the metabolic traits studied in this research is quite slower than that of body weights, that leads to a decline in meat quality. Therefore, mapping QTLs of allometric scalings and performing marker-assisted selection may be a way of improving meat quality in animal breeding programs.

On the other hand, the genetic regulation mechanisms of absolute and relative growth might be totally different. With the same dataset, we compared the QTL mapping results for relative and absolute growth of each analyzed body composition or metabolic trait (the QTL mapping results for absolute growth of body weight, body compositions and metabolic traits are shown in Supplementary Tables S1–S3), and found no identical or highly linked QTLs. After stepwise regression, two of eight metabolic traits were significantly associated with body weight in relative growth. Five QTLs (Gga 4, 6, 1, 7 and 9) affecting allometric scaling of T4 to body weight were identified in the present study, but none of them appeared in the QTL positions for absolute plasma T4 concentrations in previous studies [30,39]. Five QTLs (Gga 1 and 2) for allometric scaling of GLC to body weight were not published before either.

Inspired by the results of our research, we suggest choosing the allometric scalings relative to body weight as a new breeding target and explore methods for gene mapping analysis to improve meat quality or accelerate the genetic improvement progress of some body composition trait. Therefore, besides introducing high-quality parents for crossbreeding, we can use detected QTLs and highly linked markers of allometric growth of meat quality to assist animal breeding and promote the simultaneous growth of meat quality and body weight [40,41].

Theoretically, RRM has been used to model genetic changes of growth and developmental traits with age in plant and animals, and was able to analyze both static and ontogenetic allometry scalings. When growth and developmental traits were repeatedly measured, the RRM for ontogenetic allometry scalings could better characterize genetic changes in allometries by additionally taking into account time dependent permanent environment effects [42]. In this study, application of RRM to genetic analysis for static allometry scalings was conducted because the traits were measured only once in slaughter period. Our LASSO-RRM method was developed upon linkage analysis with markers of moderate density, which could detect QTLs for allometric scalings, but also growth and developmental traits. With high-throughput markers, our method could be easily extended to genome-wide association studies.

## 5. Conclusions

We have formulated two joint static allometric scaling models to simultaneously evaluate the allometric scalings of body compositions and metabolic characteristics to body weights of chickens, respectively, where the correlations among multiple body compositions or metabolic traits were considered. Furthermore, the QTLs for allometric scalings were mapped with a random regression model for the logarithm of body weight as the target trait taking the static allometric growth model as sub-model. By taking into account the polygenic effect estimated with pedigrees, the model we constructed had higher statistical power to detect QTLs for allometric traits.

## Figures and Tables

**Table 1:** QTLs for the allometric scalings of 3 body compositions to body weights.

Trait	QTL no. ^1^	Chr-pos. ^2^	Marker Interval	Inheritance	Effect	*t*-Value
Fat	1	2–362.5	ADL197~LEI147	additive	0.007	3.104
	2	6–73.4	LEI97~ADL138	additive	0.005	2.333
	3	27–0	MCW328	additive	0.003	2.237
	4	7–131.6	ADL109~MCW201	dominant	0.019	3.514
	5	13–65.8	ADL147~MCW244	dominant	0.016	2.211
Shank-w ^3^	1	8–65.2	LEI136	additive	0.003	2.909
	2	6–37.9	LEI97~ADL138	dominant	0.010	3.952
	3	11–325	LEI143~ADL210	dominant	0.006	2.127
Liver	1	3–325	LEI265~LEI65	dominant	0.045	1.998

^1^ QTL no. = QTL number; ^2^ Chr-pos. = Chromosome position; ^3^ Shank-w = shank weight.

**Table 2:** QTLs for the allometric scaling of two metabolic traits to body weights.

Trait	QTL no. ^1^	Chr-pos. ^2^	Marker Interval	Inheritance	Effect	*t*-Value
T4 ^3^	1	4–113.4	ADL144	additive	−0.019	2.068
	2	6–69.3	LEI97~ADL138	additive	0.027	2.949
	3	1–300.4	MCW200~ADL148	dominant	0.027	2.657
	4	7–182.5	MCW178~ADL107	dominant	0.027	2.523
	5	9–22.8	ADL136~MCW84	dominant	0.038	3.131
GLC ^4^	1	1–234.8	ADL268	additive	0.003	2.463
	2	2–70.7	MCW247	additive	0.004	3.055
	3	1–511.8	LAMP1~ADL101	dominant	−0.019	1.982
	4	1–621.2	ADL238	dominant	−0.004	2.054
	5	17–72.3	ADL202~ADL199	dominant	−0.005	2.464

^1^ QTL no. = QTL number; ^2^ Chr-pos. = Chromosome position; ^3^ T4 = thyroxine; ^4^ GLC = glucose.

**Table 3:** Information of the simulated QTLs.

QTL ^1^	Q_1_^2^	Q_2_	Q_3_	Q_4_	Q_5_	Q_6_	Q_7_	Q_8_
Chromosome	1	1	2	2	2	3	3	3
True Position	25	75	38	38	38	82	82	82
Allometric trait	Fat	Shank-w	Fat	Shank-w	Liver	Fat	Shank-w	Liver
True Effect	−0.027	0.038	−0.025	0.003	−0.054	−0.025	0.008	−0.076

^1^ QTL denotes quantitative trait locus; ^2^ Q_i_ denotes the *i*-th QTL simulated.

**Table 4:** Statistical powers and estimated QTL parameters (standard deviations) calculated with the LAASO-RRM method in simulations.

Marker Density		Q_1_^1^	Q_2_	Q_3_	Q_4_	Q_5_	Q_6_	Q_7_	Q_8_
1 *cM*	Power	54.5	84.5	15.5	5.5	83.5	14.5	12.5	98.5
	Position ^2^	24.9 (0.3)	75.2 (0.8)	37.8 (1.7)	37.9 (0.8)	38.2 (0.5)	82.0 (0.0)	81.7 (1.2)	81.9 (0.2)
	Effect ^2^	−0.028 (0.011)	0.034 (0.018)	−0.019 (0.006)	0.005 (0.002)	−0.055 (0.035)	−0.015 (0.006)	0.012 (0.005)	−0.078 (0.022)
5 *cM*	Power	35.5	48.5	12.0	3.0	53.5	11.5	7.5	91.5
	Position	25.1 (0.7)	75.2 (1.1)	38.2 (1.2)	38.5 (0.7)	38.1 (0.4)	82.4 (0.7)	83.7 (1.5)	82.2 (0.4)
	Effect	−0.026 (0.013)	0.037 (0.023)	−0.016 (0.005)	0.007 (0.001)	−0.057 (0.016)	−0.021 (0.008)	0.006 (0.002)	−0.072 (0.015)
10 *cM*	Power	14.0	30.5	9.0	3.0	43.0	4.5	7.0	81.5
	Position	25.6 (0.5)	76.1 (2.2)	38.1 (0.7)	38.7 (1.1)	38.4 (0.6)	81.7 (0.7)	81.1 (1.2)	82.6 (0.6)
	Effect	−0.029 (0.012)	0.040 (0.019)	−0.023 (0.008)	0.006 (0.003)	−0.058 (0.024)	−0.028 (0.011)	0.005 (0.001)	−0.073 (0.017)

^1^ Q_i_ denotes the *i*-th QTL simulated; ^2^ The means and standard deviations for QTL positions and effects are calculated from 200 simulations.

## Supplementary Materials

The following are available online at https://www.mdpi.com/2076-2615/11/2/469/s1. Table S1. QTL mapping for absolute growth of body weight; Table S2. QTL mapping for absolute growth of body composition traits; Table S3. QTL mapping for absolute growth of metabolic traits.
