# Supplementary material for: Genetic Association Analysis for Relative Growths of Body Compositions and Metabolic Traits to Body Weights in Broilers
Source: Animals (Basel). 2021 Feb 10;11(2):469. doi: 10.3390/ani11020469 (PMC7916405; doi:10.3390/ani11020469)
Supplement: Supplementary file 1 [file animals-11-00469-s001.pdf]

Table S1. *QTL mapping for absolute growth of body weight*

QTL no.	Chr-pos.	Marker interval	Inheritance	Additive	Dominance	t
1	1-234.8	ADL268	additive	37.31		2.66
2	1-514.3	ADL101	additive	107.73		2.55
3	1-631.9	ADL238~ROS25	additive	80.97		2.06
4	2-70.7	MCW247	additive	41.01		2.65
5	2-173.9	ADL185~ADL309	additive	95.33		2.14
6	2-426.6	ADL267~MCW27	additive	-39.21		2.55
7	2-473.0	MCW185	additive	-59.71		2.12
8	4-11.6	ADL317~ MCW295	additive	-65.81		2.13
9	5-103.0	LEI145 ~ MCW238	additive	50.33		2.24
10	6-69.3	LEI97 ~ ADL138	additive	84.30		2.69
11	1-432.6	ADL198 ~MCW177	dominance		-148.86	2.92
12	8-32.9	ABR345 ~MCW147	dominance		107.57	3.10
13	9-29.6	MCW84	dominance		72.60	3.40

Table S2. *QTL mapping for absolute growth of body composition traits*

Trait	QTL no.	Chr-pos.	Marker interval	Inheritance	Additive	Dominance	t
Fat	1	6-41.9	LEI93~LEI97	additive	1.01		3.56
Shank-w	1	1-36.8	MCW106	additive	-1.33		2.36
	2	1-237.2	ADL268~MCW313	additive	1.08		2.40
	3	1-432.5	MCW177~ADL183	additive	1.29		3.12
	4	1-500.2	LEI107	additive	-1.87		2.27
	5	1-654.9	ROS25~LEI134	additive	1.87		4.62
	6	9-90	MCW135	dominance		1.84	2.14
Liver	1	1-441.3	ADL183~LEI106	additive	2.55		2.44
	2	2-473	MCW185	additive	-4.75		3.70
	3	9-20.5	ADL136~MCW84	additive	-8.57		3.78
	4	1-423.6	ADL198~MCW177	dominance		-5.07	2.48
	5	Z-98.4	MCW294~LEI121	dominance		3.92	2.87

Table S3. *QTL mapping for absolute growth of metabolic traits*

Trait	QTL no.	Chr-pos.	Marker interval	Inheritance	Additive	Dominance	t
T4	1	3-432.9	MCW37	dominance		-0.81	3.14
GLC	1	2-416	ADL267	additive	124.54		2.89
	2	2-588.3	MCW220	additive	-99.67		2.08
	3	2-135.5	MCW239	dominance		304.01	4.30
	4	2-322.9	MCW293	dominance		170.44	2.56
